# Get the GIST: A Case Study of Detection and Treatment of Gastrointestinal Stromal Tumors

**DOI:** 10.7759/cureus.11952

**Published:** 2020-12-07

**Authors:** Mason Bennett, Joseph Lomboy

**Affiliations:** 1 Medicine, Trinity School of Medicine, Ribishi, VCT; 2 Internal Medicine, Houston Medical Center, Warner Robins, USA

**Keywords:** gastrointestinal stromal tumor (gist)

## Abstract

Gastrointestinal stromal tumors (GISTs) are soft tissue sarcomas that can occur anywhere in the GI tract. There are roughly 4,000 to 6,000 cases diagnosed in the United States annually. GISTs are often asymptomatic early on and can evade detection, occasionally resulting in malignancy. Due to their insidious growth and location, it is suspected that they are more common than currently reported. It is important to know how difficult it is to identify a GIST and the various methods to treat it in a patient. Our case presents a 62-year-old male with incidental findings of multiple GISTs during workup for kidney stones. The patient was fortunate that these tumors were detected before developing into a greater health concern and this case highlights the insidious nature with which they develop.

## Introduction

Mesenchymal tumors are soft tissue tumors affecting connective tissue. The most common system they affect is the GI tract, usually manifesting as gastrointestinal stromal tumors (GISTs) [1]. GISTs are rare, accounting for about 1% of all gastrointestinal tumors. It is suspected that sub-centimeter, non-symptomatic GISTs may be more common than currently reported [2]. However, large symptomatic tumors are still uncommon and are found in about seven in a million people [3].

GISTs arise from interstitial cells of Cajal [4]. GISTs can grow to incredible size; the largest recorded is 18.6 kg [5]. As tumors grow, so do complications, namely mass effect on the GI system and local organs, necrosis with calcifications, and increased risk of metastasis in tumors greater than 2 cm in a non-gastric location, and with a mitotic index greater than five mitoses per 50-high powered field [6]. However, it should be noted that all GISTs, regardless of size, have malignant potential.

GISTs usually do not present with specific symptoms [6]. When they do, common presentations include anemia, abdominal pain, nausea, vomiting, loss of weight or appetite, or even a palpable mass in the abdomen, and thus, GISTs are commonly incidental findings from other exams. Once they are suspected, imaging studies can be performed. Definitive diagnosis is accomplished through biopsy, with key findings including tumor morphology and mitotic count for malignancy [7]. Genetic studies may also aid in identifying GISTs, as all GISTs are positive for KIT and CD34 expression, with platelet-derived growth factor receptor alpha mutation appearing in 50% of all cases. Location is another important clue, as 60% of lesions are found in the stomach and 30% in the small intestine [1]. Finally, age and sex may increase suspicion, with 50- to 70-year-old men most frequently affected [8].

If tumors are less than 2 cm in size, non-symptomatic, or in a patient who cannot support surgery, then monitoring with endoscopic ultrasonographic examinations may be enough [6]. As with diagnosis, surgery is the treatment of choice for suspicious or at-risk tumors, although with surgery alone, 50% of patients have a recurrence of a GIST within five years [5], and so proper follow up with CT of the abdomen and pelvis every three to six months is recommended [6]. In addition, post-surgical treatment with imatinib has been shown to reduce the recurrence rate and increase the survival rate by up to 98% [9]. 

## Case presentation

Presentation and diagnosis

Our patient is a 62-year-old Caucasian man who went to a health fair at his church where abdominal sonograms were being performed. After receiving a sonogram the patient was told to see his primary care provider about possible kidney stones. The primary care physician performed an additional sonogram and confirmed kidney stones with an incidental finding of gallstones. The patient was referred out for a CT scan for workup of kidney stones. The patient underwent stent placement and lithotripsy. Soon after lithotripsy, the patient developed fever, and a CT with contrast was performed. This scan showed incidental findings of masses on the gastric fundus. An endoscopic exam was performed with a biopsy taken which showed spindle cells, and the first diagnosis of GIST was made.

Treatment

**Figure 1:**
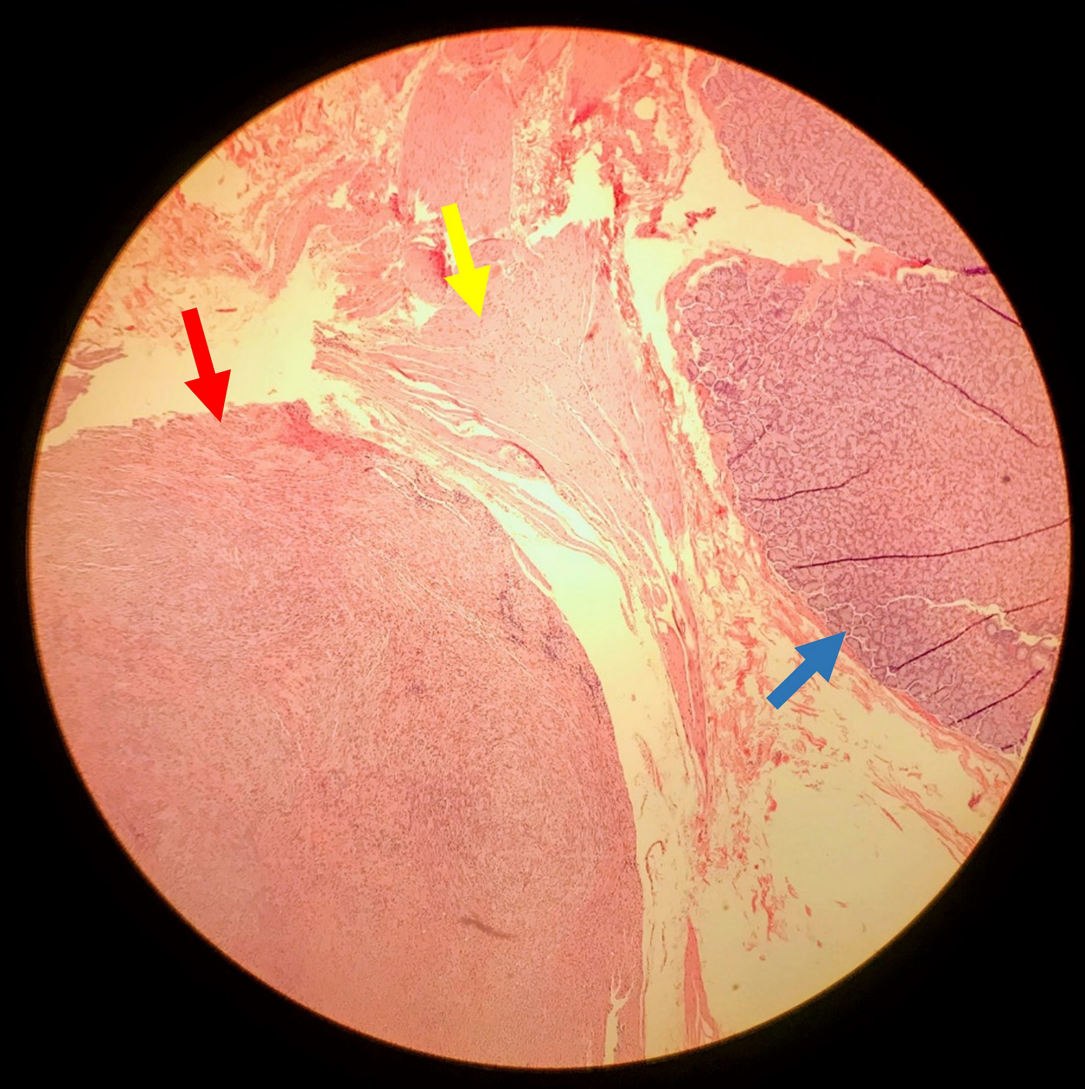
Sample from posterior tumor. Red arrow: gastrointestinal stromal tumor (GIST). Yellow arrow: muscularis propria. Blue arrow: mucosa of the stomach

The patient underwent a cholecystectomy, during which time the surgeon also removed the gastric masses. A light tan nodular mass, 4.5 x 2.2 x 1.8 cm, was removed from the posterior stomach, with histological findings as noted in Figure 1. We also obtained a light pink and tan wedge-shaped sample measuring 2.9 x 2.5 x 2 cm from the anterior stomach. Samples were sent to pathology where they showed spindle cell neoplasms composed of interlacing bundles of spindle cells with rounded to tapered nuclei. The mitotic count was low, with only one mitotic figure found in an examination of 15 high powered fields. Calcifications were seen and the edge of the tumor was well-demarcated with a thin fibrous membrane.

Follow up

The patient was sent home to recover, and roughly three weeks after the procedure returned for a kidney/ureter/bladder x-ray, which had no relevant findings. Follow up with his primary care provider showed well healing surgical incisions, improved energy and positive affect regarding his own health. We advised the patient to have follow-up visits every six months for the next five years to check for a possible resurgence of GIST. Pathology results showed benign features, and thus the patient did not require adjuvant treatment with imatinib. The patient is scheduled for a six month follow-up with his surgeon for a CT of the abdomen.

## Discussion

Seventy percent of GISTs are discovered because patients developed the symptoms mentioned earlier, namely, anemia, abdominal pain, nausea, vomiting, loss of weight or appetite. More emergent presentations are also possible, such as hemoperitoneum secondary to a ruptured GIST, a surgical emergency. Ten percent of GISTs that are discovered are found on autopsy, and 20% are found incidentally [7]. It is very likely that GIST is underestimated in the general population [2], as not every death is autopsied, not every GIST develops symptoms, and not every patient has co-morbid conditions that result in an incidental finding.

Our patient was ironically fortunate to be suffering from nephrolithiasis and cholelithiasis. Without the complication and subsequent evaluation of these stones, it is possible that his GIST would have continued to grow undetected until it reached a more dangerous stage. Since there is no routine test to screen for GIST, and not every patient is worked up for stones, those without co-morbid conditions often remain unaware until symptoms arise. Further, as noted, GISTs often present with nonspecific symptoms such as anemia or nausea. All of these factors together help to explain why GISTs may be underdiagnosed in the general population [2].

Despite this, mortality from GISTs is low, with 4,000-6,000 new cases of GISTs diagnosed annually in the United States and a five-year survival rate of 83% [10]. Given this, it is generally accepted that GISTs do not warrant routine screening. However, screening may be merited in certain populations such as those with a family history of GIST. That being said, our patient did not have a family history of GIST, was asymptomatic, and had no physical exam findings suggestive of GIST-he was never tested for mutations in these genes. This then leads us to the supposition: can and should screening for GIST be expanded? We present our patient as an example of higher risk GIST (greater than 2 cm) that benefited from incidental screening. Our patient was thankfully able to have these removed before the tumors grew and developed into a larger problem.

## Conclusions

GIST development is a quiet process that can easily elude detection. Our patient exemplifies the insidious nature of GIST progression, presenting among the 20% of incidentally detected GISTs. Despite frequent abdominal health concerns and multiple imaging tests, our patient’s tumors went undiscovered for months. Though GISTs are rare, they are still is the most common mesenchymal tumor of the GI, and if left untreated, can hamper health and develop into deadly metastasis. There are certainly others, like our patient, who are currently carrying undetected GISTs, and we suggest further research into low-risk screening may be merited.
